# Supplementary figures and images for: Common communicable diseases in the general population in France during the COVID-19 pandemic
Source: PLoS One. 2021 Oct 11;16(10):e0258391. doi: 10.1371/journal.pone.0258391 (PMC8504745; doi:10.1371/journal.pone.0258391)

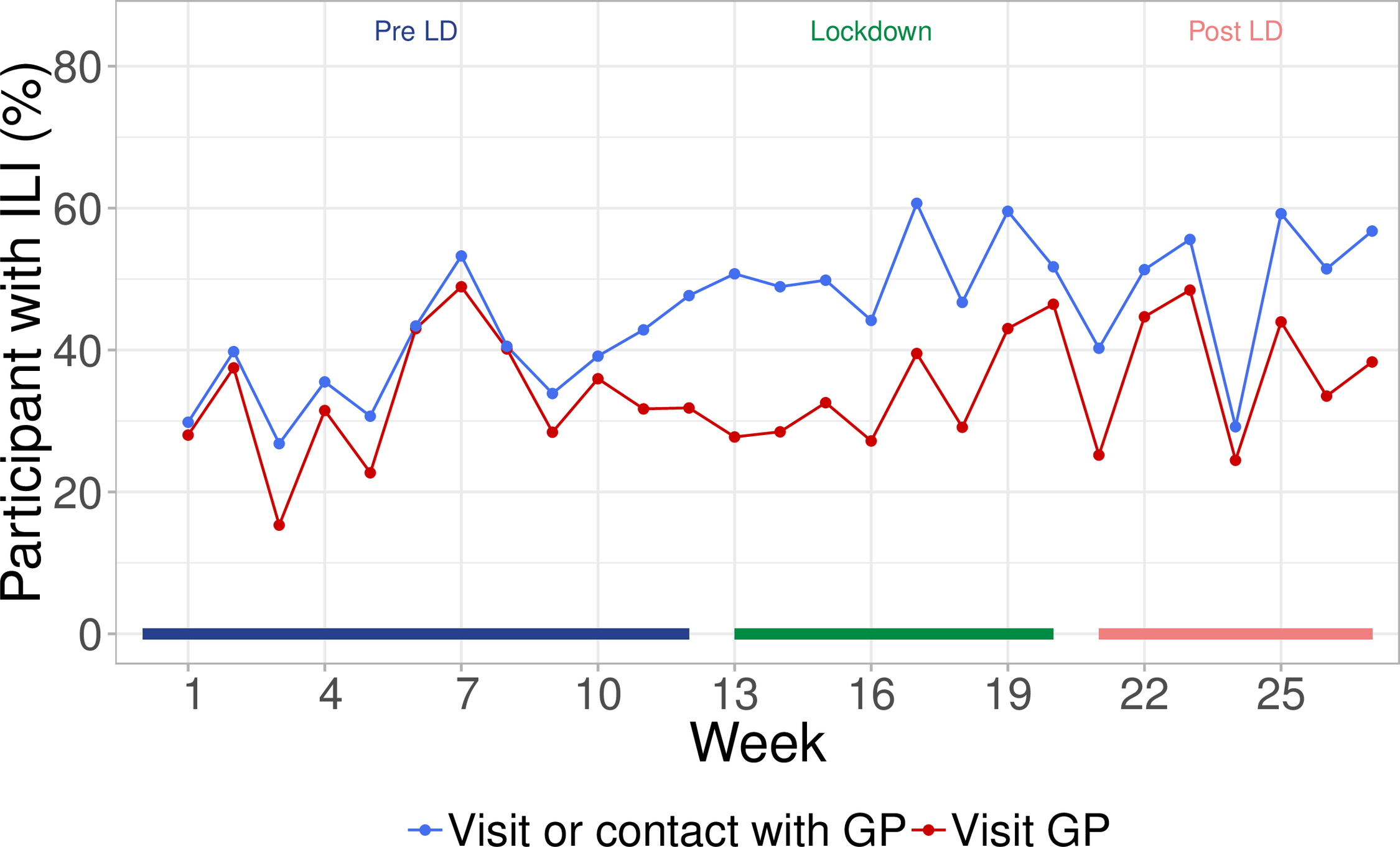

Supplement: S1 Fig — (TIF) [file pone.0258391.s001.tif]

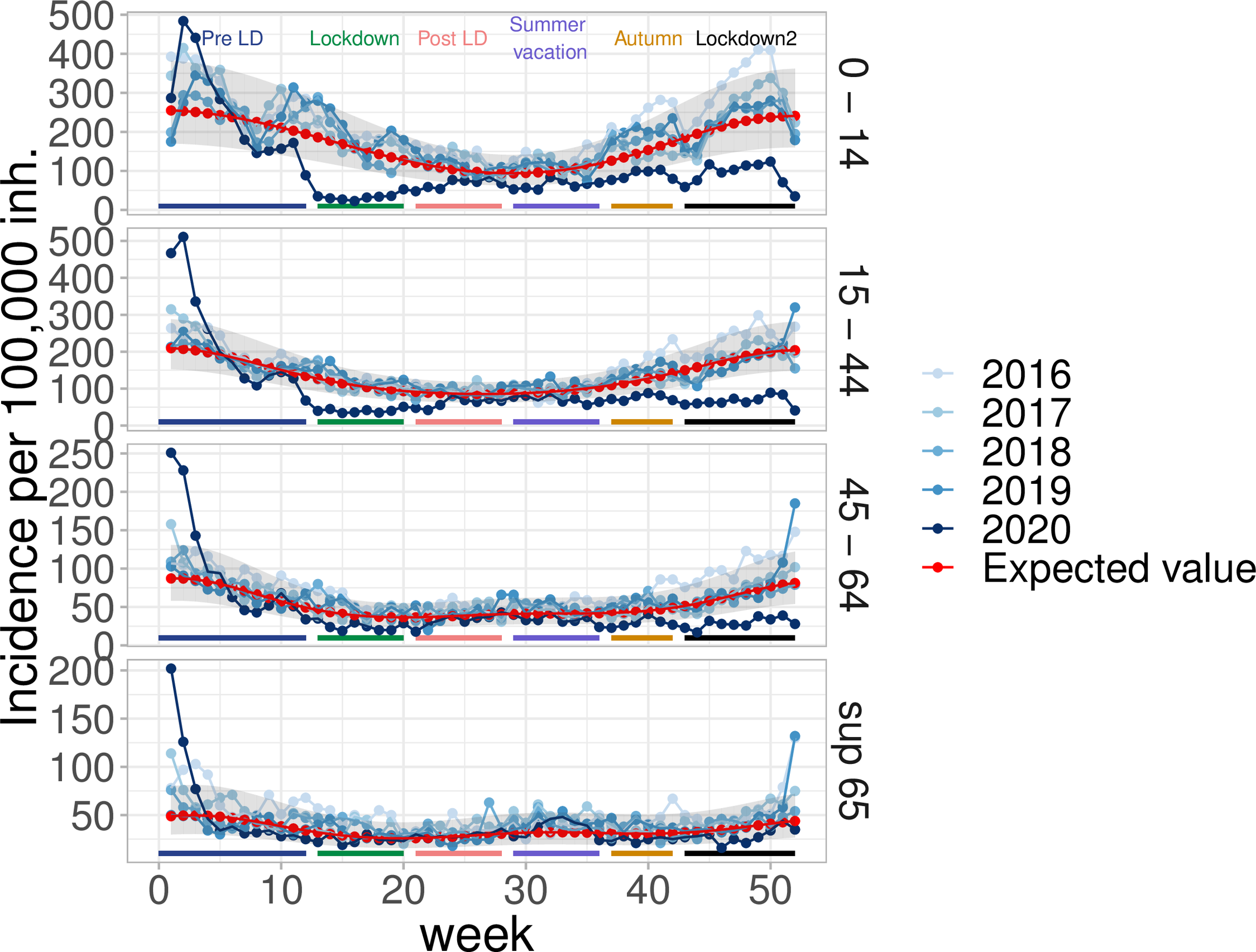

Supplement: S2 Fig — (TIF) [file pone.0258391.s002.tif]

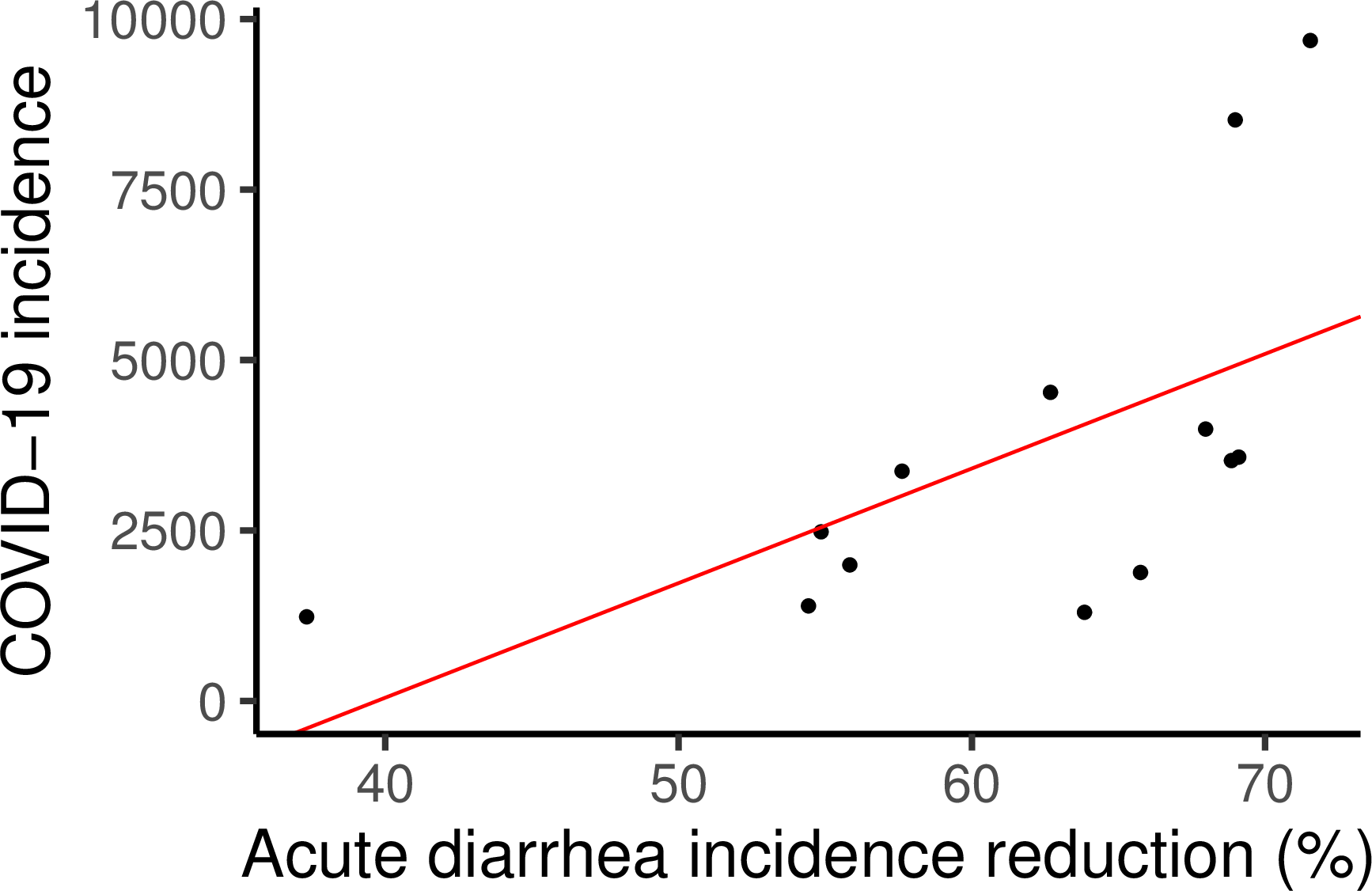

Supplement: S3 Fig — (TIF) [file pone.0258391.s003.tif]

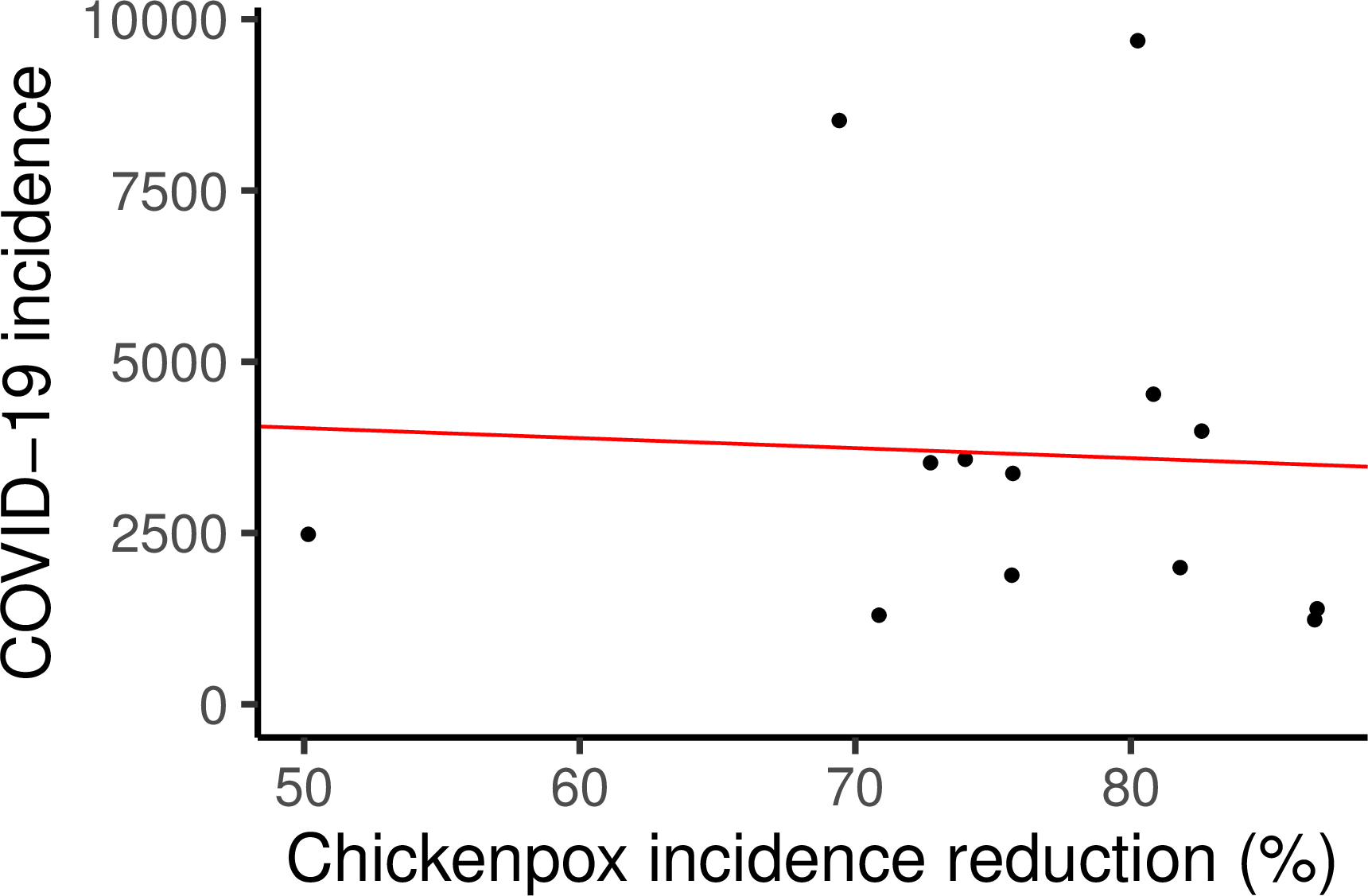

Supplement: S4 Fig — (TIF) [file pone.0258391.s004.tif]

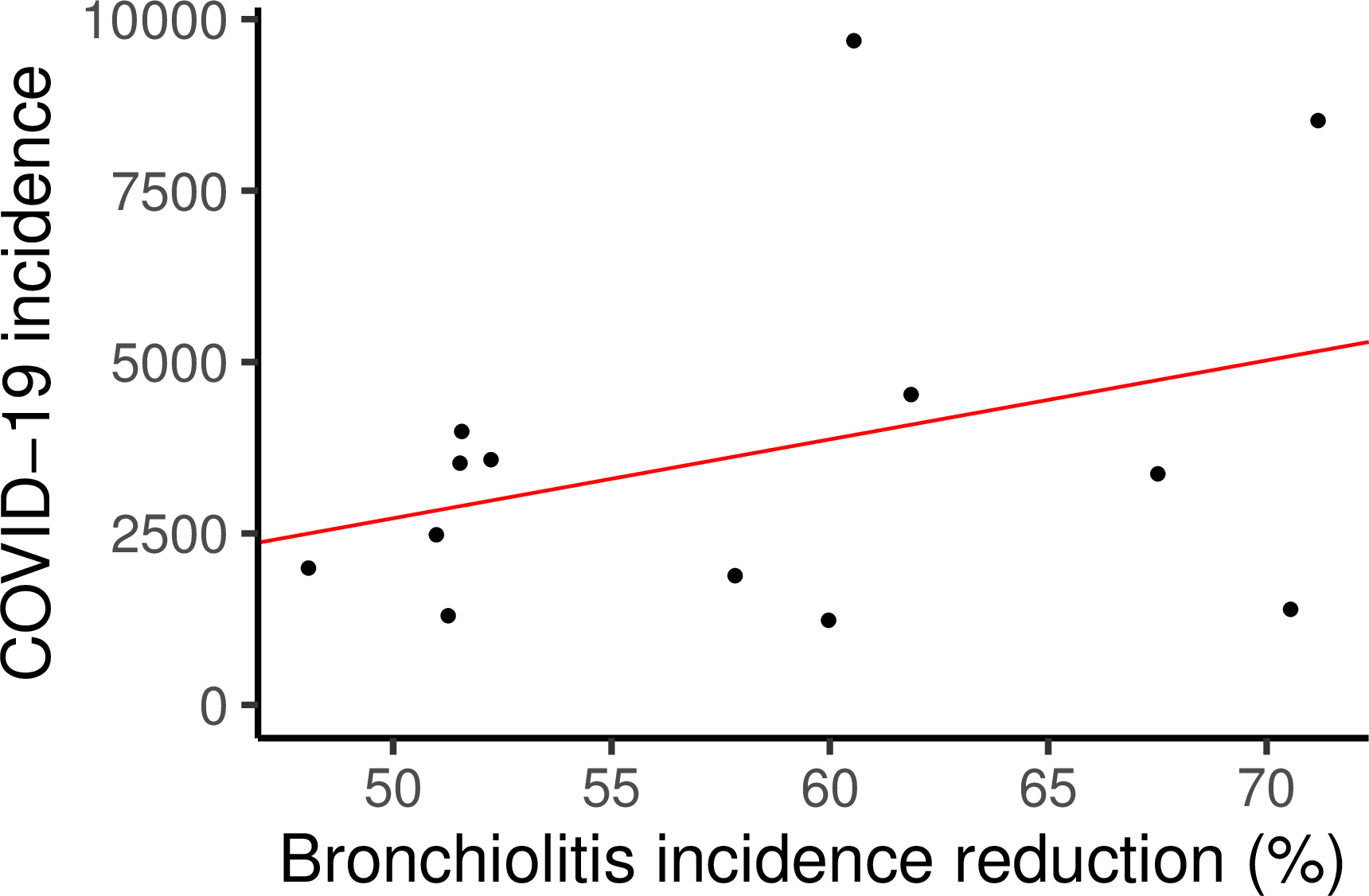

Supplement: S5 Fig — (TIF) [file pone.0258391.s005.tif]

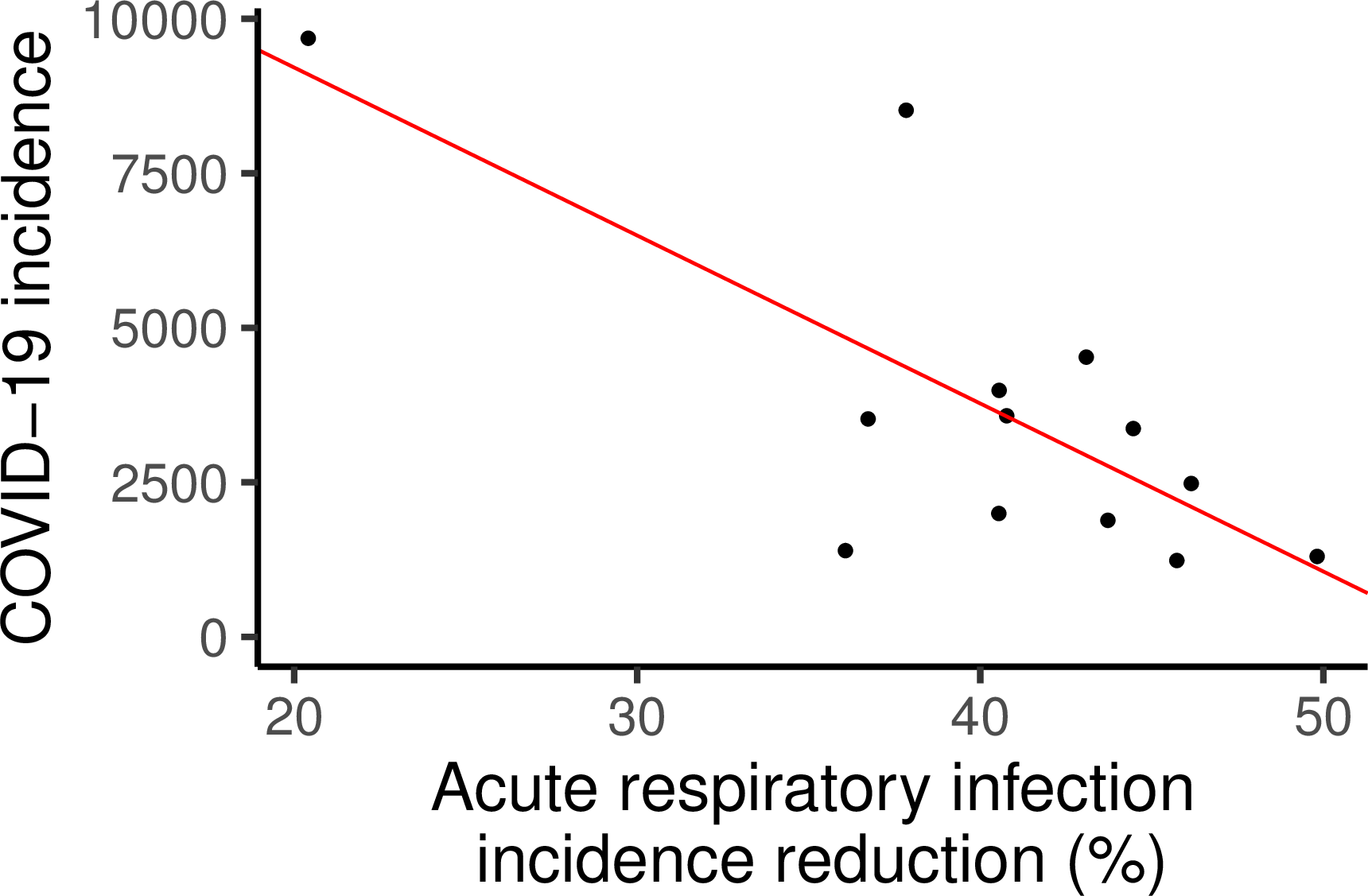

Supplement: S6 Fig — (TIF) [file pone.0258391.s006.tif]
